# Measuring calf circumference in frail hospitalized older adults and prediction of in-hospital complications and post-discharge mortality

**DOI:** 10.3389/fmed.2024.1439353

**Published:** 2024-08-06

**Authors:** Silvia Canonico, Silvia Ottaviani, Luca Tagliafico, Andrea Casabella, Alessio Signori, Marta Ponzano, Cristina Marelli, Alessio Nencioni, Fiammetta Monacelli

**Affiliations:** ^1^Section of Geriatrics, Department of Internal Medicine and Medical Specialties (DIMI), University of Genoa, Genoa, Italy; ^2^IRCCS Ospedale Policlinico San Martino, Genoa, Italy; ^3^Department of Health Science (DISSAL), University of Genoa, Genoa, Italy

**Keywords:** calf circumference, sarcopenia, frailty, mortality, in-hospital complications

## Abstract

**Background:**

Sarcopenia, characterized by muscle mass, strength, and performance decline, significantly impacts outcomes in older adults. This study aims to assess the predictive value of calf circumference (CC), in conjunction with SARC-F and hand grip, concerning in-hospital complications and post-discharge mortality among hospitalized frail older adults.

**Methods:**

A cohort of 158 hospitalized patients aged over 65 years underwent Comprehensive Geriatric Assessment and sarcopenia screening, including CC measurement. Multivariable regression analyses, adjusted for confounders, were conducted to assess predictive associations.

**Results:**

The study cohort, comprising 53% males with a median age of 86 years, exhibited significant sarcopenia prevalence based on SARC-F (85% indicating sarcopenia), hand grip strength (probable sarcopenia in 77% of males and 72% of females), and CC (sarcopenia in 83%). Multivariate analysis, adjusting for age, sex, Clinical Frailty Scale (CFS), and Mini Nutritional Assessment-Short Form (MNA-SF), demonstrated associations of CC and SARC-F with in-hospital complications, while CC also showed a significant association with reduced risks of in-hospital mortality (OR 0.441, 95% CI 0.257 to 0.754, *p* = 0.003) and 90-day mortality (OR 0.714, 95% CI 0.516 to 0.988, *p* = 0.043).

**Conclusion:**

This study provides insights into the predictive accuracy of sarcopenia screening tools on mortality in real-world hospitalized older adults with frailty. Notably, CC emerges as a robust predictor of mortality outcomes. Further research is warranted to validate and elucidate the respective contributions of CC and frailty to mortality in vulnerable populations.

## Introduction

Sarcopenia is a progressive and generalized skeletal muscle disorder characterized by reduced muscle mass, strength, and performance, associated with an increased likelihood of experiencing adverse outcomes including falls, fractures, physical disability, and mortality (1, 2).

Its prevalence ranges from 7.5% in community-dwelling older adults to 77.6% in patients undergoing rehabilitation or post-acute care (3). Up to 15% of hospitalized older adults may develop sarcopenia at discharge (4). Sarcopenia is secondary to reduced physical activity (bed rest, and physical deconditioning), multimorbidity, nutritional factors (malnutrition with or without malabsorption, gastroenteric diseases), and polypharmacy. Notably, sarcopenia is also strongly associated with frailty, a geriatric syndrome characterized by an extreme vulnerability to endogenous and exogenous stressors, resulting from age-related depletion of the body’s homeostatic reserves (5). Frailty and sarcopenia share commonalities such as muscle atrophy, dynapenia, and impaired physical function; malnutrition may be considered a harbinger between the two, ultimately leading to an acceleration of the frailty trajectory (6).

In 2019 a revised diagnostic algorithm for sarcopenia (2nd edition of the European Working Group on Sarcopenia in Older People, EWGSOP2) (7) was proposed and the SARC-F questionnaire was recommended for screening (8). SARC-F is a questionnaire consisting of five questions concerning Strength (S), Assistance with walking (A), Rising from a chair (R), Climbing stairs (C), and Falls (F). Growing evidence has underscored the role of sarcopenia screening in predicting in-hospital immediate mortality in older adults. Namely, in a Japanese retrospective study conducted on over 2.400 hospitalized over-65 patients, SARC-F score was associated with increased in-hospital mortality within 30 days (9). Similarly, a recent meta-analysis found a significant association between SARC-F and long-term mortality (<5 years) in very old age patients (10). However, Volker et al., observed high heterogeneity in the clinometric properties of SARC-F, with a wider range of sensitivity (29–55%) and specificity (69–89%) in different settings, suggesting that the addition of calf circumference (CC) could improve sensitivity, especially in community-dwellings (11). Indeed, combining calf and thigh circumferences with SARC-F is reported to enhance the diagnostic accuracy for sarcopenia in individuals aged 60 and above, providing a resource-efficient diagnostic tool (2).

CC nowadays is included in all major international consensus (7, 12, 13) and it is considered a reliable screening tool for sarcopenia and a promising prognostic indicator in older adults. Indeed, calf measurements are associated with higher readmissions and mortality rates in hospitalized older adults (14–17). In addition, measurement of the stability of CC over 4 years was associated with decreased mortality risk in a cohort of 904 community-dwelling older adults (mean age 83.8 ± 12.2) (18). Moreover, Wu and Chen demonstrated that the addition of CC to traditional measures of sarcopenia (hand grip strength, speed of gait, muscle mass) correlated with higher all-cause and CV mortality risks after a follow-up of 3 years in community-dwelling people aged 50 years or more (19).

In Japan, Ishii et al. developed a formula based on age, CC, and hand grip strength that predicts the probability of developing sarcopenia (20), which also demonstrated high sensitivity and specificity when diagnosing sarcopenia in community-dwelling adults and inpatients (21–23) or predicting long-term all-cause mortality in hospitalized older adults (24, 25).

Based on this background, the present study aims to assess the predictive accuracy of three sarcopenia evaluation tools (SARC-F, CC, and hand grip) on intra-hospital complication rate, in-hospital mortality, and mortality within 90 days post-hospital discharge in a cohort of hospitalized older adults.

## Method

This is a prospective observational study conducted on hospitalized older adults (aged over 65 years old) referred to two units (Geriatric Clinic and Transitional Care Unit) of IRCCS Hospital Polyclinic San Martino in Genoa, Italy, from January to May 2023. Patients admitted to the Geriatric Clinic ward came from the Emergency Room, while those admitted to the Transitional Care ward came from other wards of the Polyclinic and were awaiting discharge to nursing homes.

Inclusion criteria were: age 65 or older, acceptance of informed consent by the patient or patient’s legal representative. Exclusion criteria included: age under 65, lack of acceptance or withdrawal of informed consent, and patients diagnosed with end-stage diseases in need of palliative care (eg. dementia CDR 5, heart failure NYHA IV, COPD with acute respiratory failure).

Upon admission, demographic data were collected. All patients received a Comprehensive Geriatric Assessment (CGA) (26) within 72 h from admission, including Clinical Frailty Scale (CFS) (27) to assess frailty status; number of medications and ABC score to assess polypharmacy and anticholinergic burden; basic and instrumental activities of daily living (ADL and IADL) (28) to assess functional status; Short Portable Mental Status Questionnaire (SPMSQ) (29) to evaluate cognitive performance; Cumulative Illness Rating Scale (CIRS) (30) to assess multimorbidity, and Clinical Dementia Rating Scale (CDR) (31) to stratify the severity of dementia. We used the Mini Nutritional Assessment – Short Form (MNA-SF) (32) to screen for malnutrition. SARC-F (33), measurement of CC, and hand grip (HG, using a GIMA 28791 Smedley dynamometer) were used to evaluate sarcopenia. The standardized Asian Working Group for Sarcopenia (AWGS19) protocol (12) was adopted for evaluating CC, measuring the maximum value of both calves using a non-elastic tape, applying AWGS19 cut-offs: males <34 cm; females <33 cm. As for HG, we employed cut-offs from EWGSOP2 (7): males <27 kg, females <16 kg.

Hospital complication rate was documented, including incident delirium (defined as a score of 4 or higher on the 4AT test) (34), pressure ulcers, acute anemia (hemoglobin <9 g/dL), hospital-acquired infections, sepsis, catheterization during hospital stay, urinary tract infections, respiratory distress, acute heart failure, immobilization syndrome, and in-hospital mortality. In-hospital stay and discharge destination were also collected. 90-day mortality rate after post-hospital discharge was recorded through the ASL3 Genoa (Italy) county electronic database. A Complication Index was derived as the pooled rate of incidence of any of the examined complications.

The protocol was approved by the IRB (CERA N 2024-54 12/06/2024, University of Genoa, Italy) and met the guidelines of the local Governmental Agency. Patients or their proxies provided written informed consent before study inclusion. The study was performed in adherence to the Declaration of Helsinki.

## Statistical analysis

Descriptive data were reported as mean with standard deviation or median with IQR. Multivariable logistic regression was used to assess the association between sarcopenia screening tests and clinical variables. Logistic regression for dichotomic outcomes (in-hospital mortality and 90-day mortality), and linear regression for continuous outcomes (Complication Index) were used. Multivariate regression models were built using the three sarcopenia assessment methods (SARCF, CC, hand grip) and adjusted for possible confounders: sex, age, nutritional status (MNA-SF), and frailty status (CFS). An advanced statistical imputation method was applied to avoid biases from the absence of data in hand grip measurement. All reported analyses were run by RStudio (Version 2022.07) and a two-sided α less than 0.05 was considered statistically significant.

## Results

158 consecutive patients (53% male) were enrolled. As shown in Table 1, age ranged from 65 years to 101 years, with a median of 86 years (IQR 9). Upon admission, the clinical phenotype of patients was frail (median CFS 6, IQR 2) with functional decline (median ADL 2, IQR 4; median IADL 1, IQR 3), and 61% had a diagnosis of dementia (CDR >1). The most frequent complications were hospital-acquired infections (90 cases, 57%), delirium (89 cases, 56%), occurrence of pressure ulcers (51 cases, 32%); immobilization syndrome occurred in more than one third of the cases (33 patients, 34%).

**Table 1:** Clinical phenotype of the population.

	Total (*N* = 158)
Sex (*n*[%])	
Male	83 (53%)
Female	75 (47%)
Age (median[IQR])	86 (9)
Origin	
Home	141 (89%)
Residential care home	17 (11%)
CFS (median[IQR])	6 (2)
CIRS (median[IQR])	
Severity index	1.53 (0.41)
Comorbidity index	3 (2)
Polypharmacy (average[sd])	5.62 (3.33)
ACB score (average[sd])	1.35 (1.38)
ADL (median[IQR])	2 (4)
IADL (median[IQR])	1 (3)
SPMSQ (median[IQR])	5 (7)
CDR (median[IQR])	1 (2)
MNA-SF (median[IQR])	8 (5)
At risk (8–11 ppt)	87 (55%)
Malnourished (≤7 ppt)	59 (37%)
SARC-F (median[IQR])	5 (4)
≥4 ppt	135 (85%)
Hand grip (median[IQR])	14 (9)
Male* (<27 kg)	64 (77%)
Female* (<16 kg)	54 (72%)
Calf circumference (median[IQR])	29.5 (5)
Male** (<34 cm)	69 (83%)
Female** (<33 cm)	62 (83%)

CFS: clinical frailty scale; MNA-SF: mini nutritional assessment-short form; CC: calf circumference; HG: hand grip; IQR: interquartile range; sd: standard deviation. *EWGSOP2 criteria; **AWGS 19 criteria.

Regarding nutritional assessment, MNA-SF median score was 8 (IQR 5), indicating that the majority of the population was at risk of malnutrition; only 8% of the population had a normal nutritional status. As for sarcopenia screening, SARC-F median score was 5 (IQR 4); most of the analyzed subjects (85%) were suggestive of sarcopenia. At the HG test, the median value was 14 kg (IQR 9); according to EWGSOP2 criteria, 77% of male subjects and 72% of female subjects were found to have probable sarcopenia. Measuring CC, the mean value was 29.5 cm (IQR 5), meaning that sarcopenia was present in 83% of patients according to AWGS19 criteria.

By matching the data of HG and CC, a diagnosis of sarcopenia was made in 63% of our population (*n* = 99); stratifying by sex, sarcopenia was found in 66% of males (*n* = 55) and 59% of females (*n* = 44).

Hand grip strength could not be assessed in 26 out of 158 patients (including 12 men and 14 women). The multivariate statistical analysis (Table 2), adjusted for possible confounding variables showed that CC (β −0.329, 95% CI -0.477 to −0.182, *p*-value <0.001) and MNA-SF (β −0.380, 95% CI -0.564 to −0.196, p-value <0.001) were associated with the in-hospital complication rate. This result was confirmed in a sub-analysis showing that CC was the major clinical variable associated with all major in-hospital complications (see Supplementary materials); in particular, the lower the CC, the higher the risk of developing in-hospital complications and dying during hospitalization or within 90 days of discharge.

**Table 2:** Multivariate models adjusted for sex, age and frailty status.

	In-hospital mortality		Complication Index		90-day mortality	
	OR (95%CI)	*p*	β (95%CI)	*p*	OR (95%CI)	*p*
Age	1.083 (0.978–1.199)	0.127	−0.001 (−0.047–0.044)	0.959	1.064 (0.970–1.166)	0.186
Sex	10.692 (0.800–142.939)	0.073	0.322 (−0.630–1.273)	0.504	1.111 (0.118–10.439)	0.926
CFS	0.511 (0.164–1.593)	0.247	−0.028 (−0.425–0.368)	0.888	1.266 (0.526–3.045)	0.598
MNA-SF	0.657 (0.396–1.092)	0.105	−0.380 (−0.564- -0.196)	**<0.001**	1.131 (0.782–1.635)	0.514
SARC-F	2.268 (1.192–4.317)	**0.013**	0.133 (−0.059–0.325)	0.174	1.500 (0.983–2.288)	0.060
CC	0.441 (0.257–0.755)	**0.003**	−0.330 (−0.477- -0.182)	**<0.001**	0.714 (0.516–0.989)	**0.043**
HG	1.288 (0.922–1.798)	0.135	0.037 (−0.067–0.140)	0.482	1.147 (0.876–1.501)	0.313

CFS: clinical frailty scale; MNA-SF: mini nutritional assessment-short form; CC: calf circumference; HG: hand grip.Bold values mean statistically significant results (*p* < 0.05).

On the other hand, SARC-F (OR 2.268, 95% CI 1.191–4.317, *p*-value 0.013) and CC (OR 0.440, 95% CI 0.257 to 0.754, *p*-value 0.003) were associated with in-hospital mortality. Eventually, CC was associated with 90-day mortality (OR 0.714, 95% CI 0.516 to 0.988, *p*-value 0.043). Adding CIRS as an additional covariate to the multivariate model did not significantly impact the results.

## Discussion

The alarming prevalence of sarcopenia in hospitalized older adults with multimorbidity and frailty, and its association with adverse clinical outcomes, underscores the need for systematic routine screenings to overcome underdiagnosis and undertreatment. So far, there is a lack of standardization and implementation of hospital screening for sarcopenia, and a paucity of studies have investigated the association between screening tools and clinical outcomes in hospitalized older patients, with a wide heterogeneity in study designs and clinical findings (35).

To the best of our knowledge, this is the first study to assess the predictive accuracy of a series of screening tools for sarcopenia on mortality in a real-world hospitalized old population with frailty. Notably, in our hands, CC was the main determinant of 90-day mortality, while also being associated with the in-hospital complication rate and intra-hospital mortality. Similarly, also a higher SARC-F score was associated with intra-hospital mortality and a higher MNA-SF score with the in-hospital complication rate.

In line with that, Marchasson et al. showed that CC is an independent prognostic score for 1-year mortality in oncogeriatric patients submitted to chemotherapy (36). Rodrigues et al. demonstrated that CC was an accurate predictor for 36-month mortality in a cohort of 173 patients older than 60 years, undergoing maintenance hemodialysis (37). Moreover, Aliberti et al. evaluated 1-year survival of 665 acutely ill older adults and CC was the main determinant for mortality after adjustment for age, sex, race, income, Charlson comorbidity index, depressive symptoms, cognitive impairment, and unintentional weight loss (38). Recently, Li et al. observed that a 1 cm increase in CC is associated with a decrease in overall mortality in different healthcare settings (39). The recent systematic review and meta-analysis by Wei et al. confirmed the association between low CC and mortality in hospitalized adults (pooled HR = 2.63, 95% CI 1.93–3.58) (15).

A major strength of our findings is the systematic assessment of frailty and its incorporation as a covariate. Although frailty is recognized as a critical factor in predicting adverse outcomes in older adults, including mortality, CFS was not predictive of mortality. This contrasts with the study of Liao et al. (40), which showed that mortality in older adults visiting the emergency room was associated with gender, possible sarcopenia (defined by both low handgrip strength and CC), living in residential institutions and frailty based on Fried’s phenotype (41). On one hand, our study focused on advanced age groups, and the incorporation of frailty based on an accumulation model (42), although in the screening format, may have a higher likelihood to capture a broader range of frailty-related variables and their interaction with CC (43). On the other hand, the inability of the CFS to predict mortality may also be due to a ‘ceiling effect,’ as the great majority of patients had an advanced frailty status that may limit the generalization of the findings.

Furthermore, our study design is marked by the inclusion of a 90-day follow-up period, representing a clinical advancement over short-term mortality assessment (44).

Additionally, by adjusting our results for MNA-SF data, we aimed to account for the potential influence of nutritional status on the association between CC and mortality outcomes. This allows us to better understand the independent prognostic value of CC in our study population.

A relevant future development would be implementing adjustment for BMI, as suggested by Gonzalez et al. (45), or, otherwise, the adoption of normative values of CC across ages. In line with that, Martone et al. (46), through the Lookup 7+ project, showed that calf circumference decreases with advancing age in both sex. Based on these findings, a simple and practical medical device—a calf circumference measuring tape—has been developed, enabling a quick and cost-effective assessment of muscle mass. Integrating normative values for calf circumference across age groups holds promise for enhancing sarcopenia assessment and for providing a better understanding of age-related variations in muscle mass, in order to identify individuals at risk of adverse outcomes.

While CC has significant evidence as a practical tool for providing an estimate of muscle mass, there’s a gap in defining cut-off points. We used the AWGS19 threshold, higher than EWGSOP2 (31 cm), supported by Fernandes et al., who found mortality risk rising below 34.5 cm in people aged over 60.

The study has limitations, such as the limited sample size, the single hospital enrollment, and the possible inclusion of patients with specific conditions affecting CC (e.g., heart disease, venous insufficiency, or declivous edema). While patients admitted to the Transitional Care ward suffered from the most diverse diagnoses, those coming to the Geriatric Clinic ward directly from the Emergency Room usually had a chronic disease exacerbation (e.g., COPD, heart failure) or complications related to advanced frailty (ab ingestis pneumonia, pressure ulcers infections, urosepsis), and we did not systematically collect the causes for hospitalization. Hand grip strength assessment faced challenges, with some data missing due to poor compliance, altered consciousness, and cognitive impairment in certain patients. The presence of several missing data within the hand grip variable is undoubtedly a significant limitation of the study; excluded patients are highly likely to overlap with those most affected by sarcopenia, potentially leading to biased results and limiting the ability to accurately assess the relationship between hand grip strength and sarcopenia. Even among those tested, conditions like bed rest and acute illness may underestimate prehensile strength on admission. It cannot be ruled out that these factors contributed to the worse predictive performance of the hand grip test, which still remains the international gold standard for the assessment of sarcopenia.

CC could indeed represent a parameter as simple and time-saving as versatile in the hospital setting, where the performance of articulated test batteries or complex physical performance tests is prevented by the often precarious and acute condition of patients. Its easy reproducibility, even by caregivers, and, at the same time, prognostic efficacy for both short- and long-term health outcomes, makes it an useful indicator for the correct assessment of geriatric patients in multiple settings, transcending the simple evaluation of sarcopenia or nutritional status.

In conclusion, based on our findings, CC emerges as a single variable capable of being associated with three important health outcomes, bearing independent prognostic value compared to nutritional and physical performance data. Due to its ease of use, we anticipate its increasing integration into routine assessments. Its predictive value for mortality outcomes in hospitalized older adults potentially surpasses frailty in this regard. However, further research is needed to confirm and better understand the relative contributions of CC and frailty to mortality in such vulnerable populations.

## Data availability statement

The raw data supporting the conclusions of this article will be made available by the authors, without undue reservation.

## Ethics statement

The studies involving humans were approved by University Research Ethics Committee (CERA, UniGE). The studies were conducted in accordance with the local legislation and institutional requirements. Written informed consent for participation in this study was provided by the participants’ legal guardians/next of kin.

## Author contributions

SC: Writing – original draft, Data curation, Conceptualization. SO: Writing – review & editing, Writing – original draft. LT: Writing – review & editing, Methodology, Formal analysis. AC: Writing – review & editing, Supervision. AS: Writing – review & editing, Methodology, Formal analysis. MP: Writing – review & editing, Formal analysis. CM: Writing – review & editing, Formal analysis. AN: Writing – review & editing, Supervision. FM: Writing – review & editing, Supervision, Conceptualization.
