# Supplementary material for: Measuring calf circumference in frail hospitalized older adults and prediction of in-hospital complications and post-discharge mortality
Source: Front Med (Lausanne). 2024 Aug 6;11:1439353. doi: 10.3389/fmed.2024.1439353 (PMC11333210; doi:10.3389/fmed.2024.1439353)
Supplement: Supplementary file 1 [file Table_1.DOCX]

|  | **Hospital-acquired infections*** | | **Delirium*** | | **Pressure ulcers*** | |
| --- | --- | --- | --- | --- | --- | --- |
|  | OR (95% CI) | p | OR (95% CI) | p | OR (95% CI) | p |
| MNA-SF | 0.767 (0.561 – 1.048) | 0.096 | 0.731 (0.533 – 1.002) | 0.052 | 0.820 (0.551 – 1.218) | 0.325 |
| SARC-F | 1.121 (0.844 – 1.488) | 0.431 | 0.738 (0.547 – 0.994) | 0.046 | 1.061 ( 0.699 – 1.610) | 0.782 |
| CC | 0.717 (0.561 – 0 9.18) | 0.008 | 0.756 (0.592 – 0.966) | 0.025 | 0.429 (0.279 – 0.658) | <.001 |
| Hand Grip | 1.043 (0.888 – 1.226) | 0.606 | 0.856 (0.727 – 1.009) | 0.064 | 1.067 ( 0.859 – 1.324) | 0.558 |

**Table 1 – Supplementary materials**: subanalysis, adjusted by age and sex, of the predictive role towards major in-hospital complications.
